# Oleocanthal and Oleacein from Privet Leaves: An Alternative Source for High-Value Extra Virgin Olive Oil Bioactives

**DOI:** 10.3390/ijms252212020

**Published:** 2024-11-08

**Authors:** Catherine Peyrot des Gachons, Claudia Willis, Michael P. Napolitano, Abigail J. O’Keefe, Bruce A. Kimball, Louise Slade, Gary K. Beauchamp

**Affiliations:** 1 Monell Chemical Senses Center, Philadelphia, PA 19104, USA; cwillis@monell.org (C.W.); mnapolitano@monell.org (M.P.N.); bkimball@monell.org (B.A.K.); gbeauchamp@monell.org (G.K.B.); 2 Fox Chase Cancer Center, Temple University, Philadelphia, PA 19111, USA; 3 Food Polymer Science Consultancy, Morris Plains, NJ 07950, USA

**Keywords:** *Ligustrum vulgare*, secoiridoids, phenolic compounds, oleocanthal, oleacein, bioactive compounds, extra virgin olive oil

## Abstract

Current research strongly suggests that phenolic compounds in extra virgin olive oil (EVOO) are potent preventive and therapeutic agents against metabolic diseases associated with inflammation and oxidative stress. Oleocanthal (OC) and oleacein (OA) are two of the most abundant and promising EVOO phenolics. To fully establish their health-promoting efficacy, additional animal studies and human clinical trials must be conducted, but the sourcing of both compounds at gram scale, reasonable cost, and ease of access remains a challenge. Here, we describe an extraction procedure to obtain OC and OA from the common privet (*Ligustrum vulgare*), a fast-growing, semi-evergreen shrub. We show that, compared to the olive tree, in addition to its broader geographical distribution, *L. vulgare* offers the benefit of yielding both OA and OC from its leaves. We also demonstrate the necessity of providing adapted enzymatic conditions during leaf treatment to optimize OC and OA concentrations in the final extracts.

## 1. Introduction

The study of olive phenolic compounds has been steadily increasing over the years, which may be due to the accumulation of evidence of their health benefits regarding oxidative and inflammatory-related chronic diseases [1,2], which are by far the leading cause of death and disabilities in higher-income countries.

**Figure 1:**
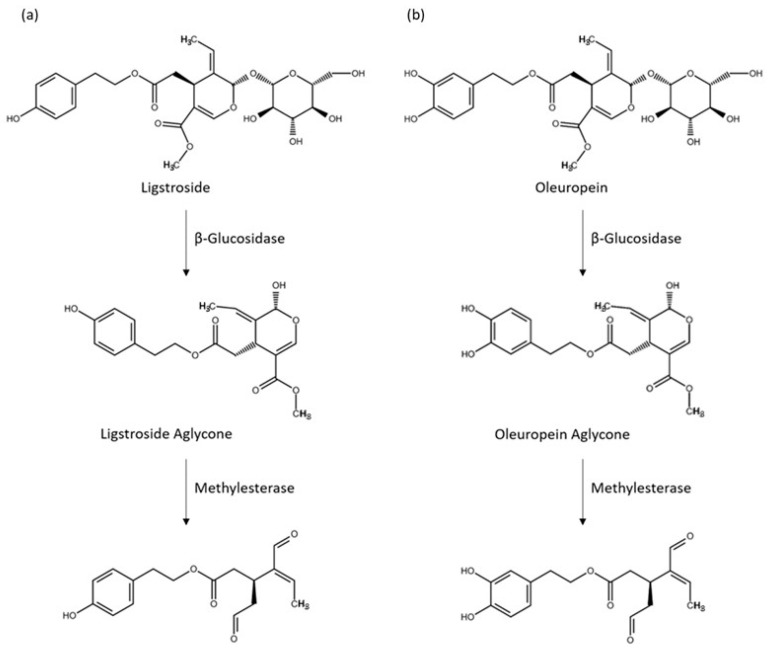
Major olive oil hydrolysis products (ligstroside aglycone, oleuropein aglycone, oleocanthal (OC), and oleacein (OA)) of ligstroside (**a**) and oleuropein (**b**), formed during olive crushing and malaxing.

The phenolic compounds from the olive tree are of diverse classes, but they are characterized by the dominant presence of the secoiridoid glucosides, especially oleuropein and, to a lesser degree, ligstroside [3]. The secoiridoid glucosides are found in most parts of the tree (i.e., fruits, leaves, stems, and roots) [4]. In olive oils, it is their hydrolysis products: oleocanthal (OC), oleacein (OA), and the aldehydic forms of oleuropein and ligstroside aglycones, which represent the major proportion of the phenolic fraction (Figure 1); they are constituents of EVOOs crucial for their shelf life stability and their perceptual properties [5], notably the positive sensory attributes, pungency, and bitterness [6,7].

Many studies have now demonstrated that these compounds also possess a wide spectrum of biological activities, such as cardioprotective, antimicrobial, neuroprotective, anti-aging, and anti-cancer effects, through the modulation of various physiological targets [8,9]. The olive oil secoiridoid derivatives hold hope for their use in the prevention and treatment of cancers, for example, and are seen as promising adjuvants in anticancer strategies [10].

Since OC and OA are often the most abundant secoiridoid derivatives in phenolic compound-rich EVOOs, and they have been shown to possess potent anti-inflammatory properties [11,12], particular attention has been directed to their bioactivities. This has led to an accumulation of evidence on their potential protective effects against inflammatory-related diseases over the last decade [13]. Although they are certainly not the only phenolic compounds contributing to the health benefits of olive oils, they may hold a particularly central place. For example, Papakonstantinou et al. [14] demonstrated that their absence substantially reduces the efficacy of an EVOO phenolic extract against a variety of cancer cell lines. In their studies, OC displayed the highest anti-proliferative and cytotoxic activities against multiple cancer lines among the five secoiridoid derivatives tested: OC, OA, oleuropein aglycone, ligstroside aglycone, and oleomissional.

The interest in health-related functions of OC and OA is, therefore, deep, but most of the published studies have been conducted in vitro, and there is only a limited number of in vivo studies. The confirmation of their beneficial effects on human health requires additional in vivo and clinical studies [15], but this research is hindered by the sparse availability of pure OC and OA [16]. Important progress has been made on the isolation of both compounds from EVOO [17], but only 2% of olive fruit phenolic compounds are estimated to be recovered into the oil phase, and olive oils rich in secoiridoid derivatives, as well as the fruits from which they are produced, are economically precious, and their access is limited. Therefore, another approach has been to develop innovative extractions of olive oil production by-products [18]. The exploitation of such waste materials is desirable to accelerate efforts in developing more sustainable food systems, but it must overcome the dynamic aspect of olive oil production, with continuing physical, enzymatic, and chemical reactions of the constituents, leading to loss or alteration of the bioactive compounds [19].

Chemical synthesis is often an excellent route to obtain large amounts of a compound. Martins et al. [20] published a review on the syntheses of ligstroside, oleuropein, OC, OA, and analogues and clearly demonstrated their potential, but also the challenges they pose, notably because of the enantiomeric forms of these compounds. As a result, there are not many syntheses described for those compounds, compared with other natural compounds [20], and to date, they are not widely used. Semi-synthetic protocols, combining efficient extraction of precursors with enzymatic catalytic conversions, are seen by some as more efficient and economical alternatives to total synthesis [21], but the use of enzymes is often difficult for scaled-up productions. Moreover, although large amounts of oleuropein can be retrieved from olive material, the supply of ligstroside is much more limited. The solid acid-catalyzed one-step synthesis of OA from oleuropein, developed recently [22], eliminates the use of additional enzymes. While seemingly a good option for OA production, due to the abundance of its precursor oleuropein, there is still the limiting factor of ligtroside scarcity for a similar OC production.

Considering the difficulties of obtaining large amounts of both OC and OA from olive plants and products, it is important to recognize that the secoiridoid phenolics, oleuropein and ligstroside and their relatives, are not restricted to the *Olea* genus (olive tree), as they also occur in other genera belonging to the *Oleaceae* family, which includes *Fraxinus, Syringa*, *Ligustrum*, *Jasminum*, *Osmanthus,* and *Phillyrea* [23]. In a search for additional sources of those compounds, we turned our attention to these other genera. Giebel et al. [24] promoted the leaves of *Ligustrum vulgare* as facile access to oleuropein, arguing that it is found in high amounts, and unlike the olive tree that only grows in Mediterranean-like climates, *L. vulgare* (or common privet in English) is adaptable to different climates. Common privet is one of the most grown plants in Eurasia and is commonly found in the US. Their leaves are also well known in historical Mediterranean medicine, especially for their oropharyngeal anti-inflammatory effects, and sparse ethnobotanical studies have reported their contemporary utilization in a few isolated Southern European areas for their anti-inflammatory, antirheumatic, diuretic, and hypotensive properties [25]. In recent years, researchers have shown that *L. vulgare* leaf extracts not only contain oleuropein but present small amounts of OA and OC [26]. We hypothesized that *Ligustrum* leaves could provide larger amounts of both OA and OC than reported, if the leaves were treated and extracted in conditions more favorable to enzymatic hydrolysis, and be used as an alternative source to olive materials. The goals of our work were, therefore, to survey *Ligustrum* leaves in the Eastern USA to determine the potential for their use as a source of OA and OC, and to develop an optimized method to obtain extracts rich in those two compounds.

## 2. Results and Discussion

**Figure 2:**
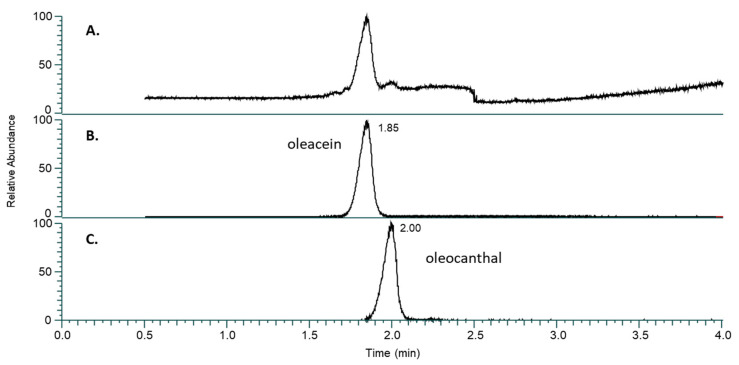
(**A**) Total ion current chromatogram across the 3.5-min acquisition with scan range *m*/*z* 50–700. (**B**) Extracted ion chromatogram (XIC) for oleacein (RT 1.85 min, *m*/*z* 319.1184). (**C**) XIC for oleocanthal (RT 2.00, *m*/*z* 303.1238), both generated with a 3-ppm tolerance. The relative abundance of (**A**–**C**) is normalized to their respective chromatograms.

**Figure 3:**
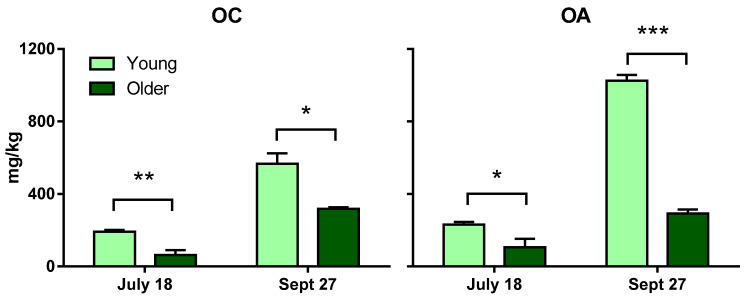
OC (**left**) and OA (**right**) amounts, expressed in mg/kg of leaves, in older leaves (dark green) and younger leaves (light green) of *Ligustrum vulgare*, at two time points during the summer (July 18) and fall (September 27) of 2022. Each bar represents the mean of three repetitions ± SEM. Statistics: unpaired *t*-test with Welch’s correction, * *p* < 0.05; ** *p* < 0.005; *** *p* < 0.0001.

We first assessed the reproducibility of our general procedure to quantify OC and OA in extracts of privet leaves by UHPLC/HRMS (see the Section 3 for leaf collection and method details, as well as Figure 2 for corresponding chromatogram). In brief, fresh privet leaves were blended with cold water buffered at pH 8 in a grinder for 30 s. The leaf slurry was filtered and then centrifuged to separate leaf debris, and a 50:50 ethyl acetate:hexane mixture was added in a 1:1 *v*/*v* ratio and stirred for 10 min. The organic phase was separated from the aqueous phase, evaporated to dryness, and then resuspended in 1 mL of acetonitrile for analyses by UHPLC/HRMS. This protocol was used for Table 1 and Table 2, and Figure 3.

In total, 12 extracts were produced and analyzed (Table 1). The results show a good reproducibility of the extraction method, with a relative standard deviation (RSD) of 11.87% for OC and 14.97% for OA. Since a portion of the variance likely comes from the leaves themselves, these values represent the minimum of experimental RSD.

One of the potential benefits of the described method is the rapid detection and assessment of recoverable OC and OA levels in leaf extracts. The procedure stands out for its simplicity, cost-effectiveness, and speed, as it does not necessitate any leaf treatment before extraction. In the previous experiment, we demonstrated the satisfactory reproducibility of the method when applied to the same lot of leaves. We next wished to establish the capability of the method to effectively distinguish between lots of leaves freshly collected from genetically different plants and extracted following the same protocol. We investigated the intra- and inter-variance in OC and OA leaf-extract concentrations in two privet species: *L. vulgare* (or common privet) and *L. ibolium* (or north privet). Both species are popular bushes, easily accessible, and mostly used for privacy hedging.

Leaves from each individual plant were collected (see Section 3) and extracted separately, and they were analyzed by UHPLC/HRMS (Table 2). There was substantial variability in OC and OA concentrations between plants of the same species, with a coefficient of variation around 20 to 30% for *L. vulgare* leaves, and a higher coefficient of variation, from 50 to 75% for *L. ibolium* leaves (Table 2). Nevertheless, the two species were clearly distinguishable with *L. vulgare* plants consistently producing leaf extracts, with higher concentrations of OC and OA compared to *L. ibolium* plants (*t*-tests indicated *p* < 0.0001 for [OC] and *p* = 0.0004 for [OA]). Interestingly, although OA is present in *L. ibolium* extracts, its level is noticeably low.

The observed intra-species variability in OC and OA levels likely reflects the impact of multiple factors, including micro-environmental factors known to influence phytochemical production such as light exposure, micro-temperature, water and nutrient availabilities, soil composition, plant-to-plant communication, or phytosanitary conditions, in addition to the variation due to leaf collection, as seen in Table 1.

There are limited points of comparison in the literature for OC or even OA concentrations in *Ligustrum* leaf extracts from different species. The most abundant and highly studied secoiridoid in the *Oleaceae* family leaves is oleuropein, the glycosylated precursor of OA, although, to our knowledge, there is no study comparing oleuropein amounts in different *Ligustrum* species. Thus, we must turn to olive trees for comparison. Several studies investigating phytochemical contents, including oleuropein, in leaf extracts from different cultivars of *Olea europaea* L. (olive tree) show large variations of this compound [27]. To learn about the influence of the genetic factor on OC and OA levels, one must look at studies of olive oil rather than olive leaves. These studies clearly demonstrate that genetics play an important role, along with processing and environmental factors. Some cultivars provide olive oils particularly rich in OC and OA [28]. For example, in a study by Miho et al. [29] evaluating the interaction of cultivar and interannual factors, OC concentrations in the olive oil were shown to be mainly influenced by the cultivar factor (60%), whereas the cultivar was the secondary factor for OA content variation but still explained about 40% of the variance. Thus, our results on privet leaves, showing a strong effect of the species factor on OC and OA contents in the extracts, are in alignment with results obtained elsewhere with olive materials. They also support the use of our method as a rapid and reliable way of assessing OC and OA potential in privet leaves.

### 2.1. Younger Leaf Extracts Provide Higher Levels of OC and OA than Extracts of Older Leaves

As a step to investigate optimal conditions to obtain large amounts of OC and OA, we next examined whether levels of OC and OA differ between extracts of younger leaves and extracts of older leaves. The leaves were collected, once in the summer and once in the fall, from *L. vulgare* plants (see Section 3). Three separate extractions were conducted for each lot of leaves, still following the same protocol and analyzed by UHPLC/HRMS for OC and OA concentrations.

As shown in Figure 3, extracts from younger *L. vulgare* leaves were richer in OC and OA than from older leaves, and this was true for both collection dates. That is, OC content in younger leaf extracts was statistically greater than that in older leaves on 18 July (*p* = 0.0015) and 27 September (*p* = 0.0166). Similarly, OA content in younger leaf extracts was also statistically greater than that in older leaves, for both dates, with *p* = 0.012 and *p* < 0.0001, respectively.

Some results consistent with our privet data have been reported for olive plant materials. Ryan et al. [30] looked at phenolic compound content, including oleuropein and OA, in the development stages of different tissues of Hardy’s Mammoth, an Australian olive cultivar. New- and old-season leaves were monitored during the olive maturation period. New-season leaves, still soft, corresponded to leaves above the fruiting zone and toward the extreme tip of the selected shoot. Old-season leaves encompassed the leaves that grew between and beyond the fruiting zone and toward the tree trunk. Leaves were frozen in liquid nitrogen in the field and then freeze-dried. Dried leaf matter was blended with methanol:water (50:50 *v*/*v*), and the solution was left to stand for 30 min at ambient temperature and then filtered. In those conditions, new-season leaf extracts had higher amounts of OA than old-season leaf extracts. One mechanism underlying the finding of higher amounts of OC and OA in younger privet leaves might be a higher level of β-glucosidase deglycosylating oleuropein and ligstroside. Koudounas et al. [31] observed, by Northern blot analysis, higher accumulations of OeGLU mRNA (corresponding to the relevant β-glucosidase) in young (expanding) olive leaves than in mature (fully expanded) leaves. They hypothesized that proliferating tissues may be more vulnerable to common pests and, thus, may have a greater demand for the oleuropein-mediated defense mechanism.

Our data from 2022 indicated that the leaves from the September collection provided higher amounts of OC and OA than did the leaves from July (Figure 3), but of course, it would be necessary to monitor regular time points over the full growing season to make conclusions about seasonal variations in OC and OA potential in *L. vulgare* leaves.

Taken together, the data presented in Table 2 and Figure 3, regarding content variation of OC and OA from leaf extracts obtained with our rapid method, demonstrate consistency and a good agreement with previously published works and can thus be a helpful tool in accumulating large sets of data on oleuropein and ligstroside hydrolysis products in plant materials. The results also suggest that the species factor of privet plants has a stronger influence on OC and OA content in the extracts than does the leaf-collection time during the season or the leaf-developmental stage. Additional studies will be needed to confirm and deepen these observations.

### 2.2. Optimized Enzymatic Hydrolysis Conditions Have Major Effects on OC and OA Concentrations in Final Privet Leaf Extracts

The primary goal of our work with privet leaves was to determine whether they represent a viable alternative source of OA, and in particular, OC, compared with limited and precious phenolic-rich EVOOs. We thus proceeded to investigate variables that could enhance further OC and OA yields. We chose to evaluate three variables (leaf drying, pH, and maceration time), keeping all other conditions the same as in the protocol previously described. The choice of the three variables was based on the following considerations. It is believed that OC and OA are not freely present in the *Oleaceae* plant parts in any significant amounts but are produced via enzymatic transformations from oleuropein and ligstroside, respectively [32], when the tissues are under physical attack (Figure 1). Processing conditions of the leaves most suitable to these enzymatic reactions should, therefore, impact OC and OA generation, starting with the leaf treatment. In general, the treatment of plant material aims to prevent the degradation of bioactive compounds, as well as the growth of fungi and bacteria, for subsequent storage. Common methods of preparing plant material include drying, freeze-drying, microwave vacuum drying, enzymatic processes, and fermentation [33]. Plant material can also be processed as untreated (fresh). Currently, the plant-extraction industry overwhelmingly uses drying, and most laboratories extracting or studying olive secoiridoids also start their extraction with dry material. Plant material drying is primarily aimed at inhibiting metabolic processes. Water in the plant material plays roles critical for plant enzymes to function properly [34]. Since we want to promote the enzymatic conversion to OC and OA, starting with dry leaves might not be the optimal approach. Similarly, grinding the leaves straight into hot water or a mixture of methanol and water or other solvents, as is commonly done, is most likely going to inhibit enzyme activities. For these reasons, we chose to grind the leaves in cold water so that the leaf slurry would reach a temperature around 20 °C, but not higher, under the heating generated by the grinding before transitioning to compound extraction with solvents. Finally, both the pH of the aqueous medium and the holding time after grinding are also expected to impact OC and OA enzymatic conversion and, thus, their contents in the final extracts. To assess the effects of these three factors (leaf drying, pH, and holding time), a 2 × 2 × 3 experimental design was conducted: undried versus fully dried, pH 8 versus pH 6 and t = 0, 15, and 60 min (see Section 3). Three separate extractions were performed for each condition.

**Figure 4:**
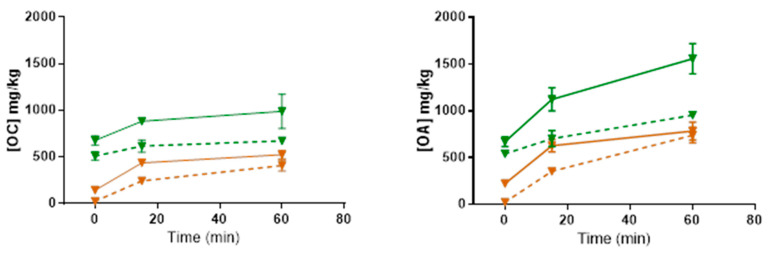
OC and OA concentrations (in mg/kg of fresh leaves) in extracts from undried (green) or fully dried (brown) *Ligustrum vulgare* leaves, collected on 30 May 2023, ground in aqueous solution at pH 6 (solid lines) or pH 8 (dashed lines). Leaf slurries had different holding time (0, 15, and 60 min) before solvent extraction. Each point represents the mean of three repetitions ± SEM.

The data from the experimental design are presented in Figure 4. The results indicate that extracts obtained with undried leaves present higher concentrations of OC and OA, regardless of the pH or the holding time. The data also show that both pH and time have an impact on the final OC and OA concentrations. Overall, the longer the contact time, the more OC and OA were present. A multifactorial ANOVA of the three independent factors on OC concentration determined that all the factors (water content (WC), pH, and time) had highly significant effects (*p* = 7.00 ×10^−11^; *p* = 7.13 ×10^−5^ and *p* = 7.97 × 10^−6^, respectively), but none of the interactions between the factors were statistically significant, indicating no combined effects on OC concentration in the extracts. The WC, pH, and time factors were also highly significant to OA concentration (*p* = 1.82 × 10^−9^, *p* = 1.56 × 10^−5^ and *p* = 3.91 × 10^−10^, respectively), but the combined effects of WC × pH × time were significant to OA concentration in the extracts (*p* = 0.022). Therefore, within the parameters studied, grinding the leaves undried at pH 6 and with a longer contact time resulted in more OC and OA in the extracts. It also shows that, under those conditions, the contact time has a stronger effect on OA concentration than on OC concentration, such as at t = 0 min, OA/OC ratio = 0.99, and at t = 60 min, OA/OC ratio = 1.57. The percentage of OC+OA, based on the total mass of the dry extract and the quantitative values obtained by UHPLC/HRMS, was calculated at 72% *w*/*w* (undried leaves, pH 6, 60 min holding time).

A previous published work [35] comparing phenolic extracts obtained from fresh and dried olive leaves observed that extracts from dried leaves presented higher levels of phenolic compounds, including a higher level of oleuropein, than did the extracts from undried leaves. Those authors attributed the lower level of oleuropein in undried leaves to the conversion of this compound into other compounds via β-glucosidase activity. Despite those experiments being conducted in conditions unfavorable to efficient metabolic conversion of oleuropein, it is likely that it was indeed partly converted. Another study [36] showed that storing fresh olive leaves for 22 h at 37 °C in closed plastic bags caused OA content to rise, with a corresponding fall in oleuropein content likely due to the softening of the leaf tissues in the moist atmosphere, allowing enzymes and substrate to come into contact. Thus, their results are aligned with ours. The proposed metabolic pathways for OC and OA production from ligstroside and oleuropein involve two enzymes: a methyl esterase and a β-glucosidase [28] (Figure 1). The optimal pH for olive β-glucosidase, with oleuropein as a substrate, is reported to be around 5 to 5.5 [37], while the esterase activity displays an optimal pH between 7 and 8 [38]. Based on our results demonstrating higher OC and OA concentrations at pH 6, the β-glucosidase activity might be a more limiting factor in OC and OA production in *L. vulgare* leaf extracts than the esterase activity. This could mean that, at pH 6, there is sufficient esterase activity for the amount of substrate present, or that this step is not fully required. Indeed, it is possible that at least a portion of OA and OC is produced from direct deglycosylation of demethyloleuropein and demethylligstroside already present in the leaves. Demethyloleuropein is another secoiridoid glucoside often present in large quantities in *Oleaceae* plants, and demethylligtroside has also been reported in leaves of the *Oleaceae* family [39].

Czerwinska et al. [40] showed that in extracts of *L. vulgare* dried leaves, OA was neither present in ethanolic extracts and decoctions nor, in infusions prepared at high temperatures. These are all conditions in which enzymatic reactions are inhibited, but the compound was found in room temperature aqueous extracts, with a concomitant decrease of oleuropein concentration. Their finding also aligns well with ours, although these authors did not find, or look for, OC in their studies.

An alternative hypothesis to explain the lower OA and OC levels obtained at pH 8 could be that polyphenols have lower stability at higher pH values with the formation of quinones. However, we do not believe this is the case since we did not observe any new spots that could correspond to quinones, in the pH 8 extracts, during the TLCs we routinely perform for checkup under UV light. Other chemical reactions occurring at both pH values cannot be ruled out without further investigation.

Here, we have demonstrated that high amounts of OC, along with OA, can be retrieved from *L. vulgare* leaves. However, the treatment parameters of the leaves, before liquid/liquid extraction, are critical since amounts of OC and OA in the final extracts can vary by a factor of at least 10. In the case presented here (Figure 4), only traces of OC or OA were found in the extracts obtained from dried leaves, crushed at pH 8, with no holding time, but extracts obtained from the same batch of leaves treated under different conditions resulted in high amounts of both compounds. Any factors limiting the activity of the enzymes hydrolyzing OC and OA precursors will negatively impact the final content of OC and OA in the extracts. Because many different extraction protocols are used in the study of secoiridoid derivatives, such as OA, in olive and other *Oleaceae* plant materials, all with different levels of enzymatic inhibition, the literature on the topic is confusing and often contradictory, as Termentzi et al. [41] have previously highlighted. We suggest that optimization, such as we have documented here, may help to resolve some of these published contradictory results.

### 2.3. L. vulgare Leaves Serve as a Rich Source of Both OC and OA Compared to Other Ligustrum Species and Olive Leaves

As previously described (Table 2), we observed lower OC and OA concentrations in extracts obtained from leaves of *L. ibolium* (North privet), as compared to extracts from leaves of *L. vulgare* (common privet). Here, we reassessed OC and OA concentrations in a series of extracts obtained using the optimized parameters for OC and OA production determined from the experimental design described in the previous section (i.e., undried leaves, ground at pH 6, with 60 min holding time). We compared extracts made from leaves of *L. vulgare*, *L. ibolium,* and the latter’s parent species *L. obtusifolium* and *L. ovalifolium*, as well as an extract from leaves freshly picked from potted *Olea europeae* Koroneiki trees (see Section 3).

**Figure 5:**
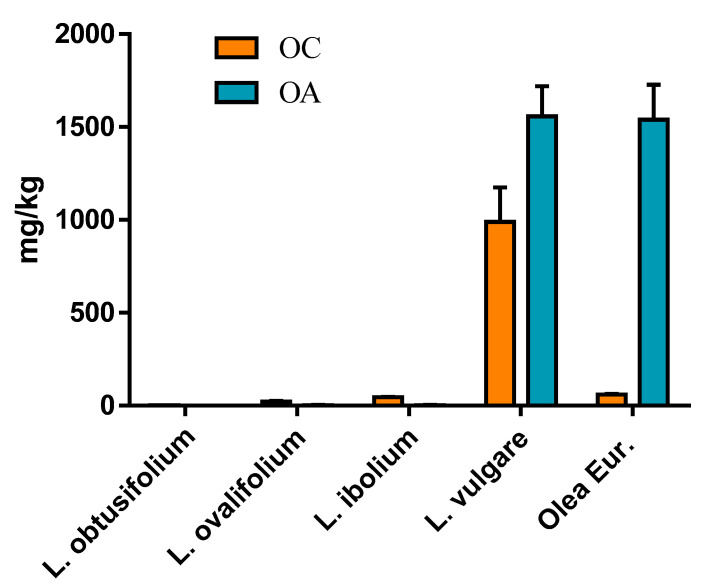
OC (orange) and OA (blue) amounts, expressed in mg/kg of leaves, in leaves of four *Ligustrum* species (privets) and *Olea europeae* Koroneiki (olive). Leaves were collected in June 2023 in the USA mid-Atlantic region. Each bar represents the average of three extracts, each obtained from an individual plant, ±SEM.

Among the privet species investigated in this work (Figure 5), *L. vulgare* leaves were the best source of OC and OA, providing 990 and 1560 mg/kg, respectively. The Koroneiki olive leaf extracts had a level of OA very similar to that in the *L. vulgare* extracts but presented a much lower content of OC at only 60 mg/kg. The data confirmed the low levels of OC and OA found in *L. ibolium* leaf extracts, at 45 and 3.6 mg/kg, respectively. Since the leaves for this experiment were collected in June, and the leaves for the extracts in Table 2 were collected in September, the low compound levels of the north privet plants appeared to be relatively independent of the phenological stage. Another interesting observation is the fact that one of its parents, *L. ovalifolium*, presented similarly low levels, with a comparable ratio OC/OA (OC = 23 and OA = 2.5 mg/kg), whereas the other parent, *L. obtusifolium*, had no noticeable amount of either OC or OA. This last observation was surprising because early work from Konno et al. [42] indicated that oleuropein makes up 3% of the wet weight of *L. obtusifolium* leaves, and that an oleuropein-specific β-glucosidase from their organelles activated the formation of an α,β-unsaturated aldehyde, which we can now assume was OA. Since the work by Konno and colleagues was performed with plants grown in Japan, and *L. obtusifolium* originated from East Asia, it is possible that some *L. obtusifolium* plants, naturalized in the USA, underwent mutations in the secoiridoid pathway, leading to the observed absence of OC and OA in our extracts. Thus, it would be compelling to compare the genomes of *L. obtusifolium* plants in East Asia and in the USA.

The direct comparison of *L. vulgare* extract with an extract from Koroneiki olive leaves highlights the particular interest of *L. vulgare* leaves, which, unlike olive leaves, can provide high amounts of both OC and OA. Although we cannot generalize our finding from one olive leaf sampling, to our knowledge, the presence of OC in meaningful amounts in olive leaf extracts of various cultivars has not been reported [30,43], except by Paiva–Martins et al. [44]. In fact, Papakonstantinou et al. [14] based their quick selective extraction of OA on the idea that there is no OC in olive leaves (they used Kalamon leaves). The amount of OA they obtained was reported to be 9.5 g from 1 kg of olive leaves with a water content under 10%. This appears in line with the amount of OA we found in *L. vulgare* leaves, 1.5 g, from 1 kg of undried leaves at about 70% water content. Czerwinska et al. [26], who studied the inhibitory effects of *L. vulgare* leaf extracts on the development of neuropathic pain, reported the presence of 20 mg of OC/kg solid leaf and 60 mg of OA/kg solid leaf in their extracts, values which are well below what we obtained with the method described here.

## 3. Materials and Methods

### 3.1. Chemicals

Sodium bicarbonate (NaHCO_3_) and sulfuric acid (H_2_SO_4_) were purchased from Sigma–Aldrich (St. Louis, MO, USA). Ethyl acetate (EtOAc), hexane, vanillin, acetonitrile (ACN), water, and Optima-grade formic acid (FA) were purchased from Fisher Scientific (Hampton, NH, USA). Ethanol (EtOH) was acquired from Decon Labs, Inc. (King of Prussia, PA, USA). Oleocanthal (95% purity) and oleacein (93%) were purchased from PhytoLab GmbH & Co. KG (Vestenbergsgreuth, Germany).

### 3.2. General Privet Leaf-Extraction Protocol

Leaves (33 g corresponding to ~10 g of solids) were blended with cold water (80 mL at 5 °C), buffered at pH 8, in a KitchenAid grinder (BCG1110B Blade Coffee Grinder) for 30 s. The leaf slurry was scraped onto a nylon cloth and held in a metal strainer atop a 500 mL beaker, where it was wrung and compressed to collect the filtrate with minimal leaf debris. Rinsing of the leaf slurry with buffered water was repeated until 75 g of filtrate was collected, and the temperature of the filtrate was recorded. Next, the filtrate was centrifuged, using 3200× *g* for 10 min (TOMY CAX-571 centrifuge, Tokyo, Japan), to separate remaining leaf debris from the filtrate. The filtrate and a 50:50 EtOAc:hexane mixture were added to a flask in a 1:1 *v*/*v* ratio and stirred for 10 min. The resulting blend was then centrifuged, using 3200× *g* for 10 min. The upper solvent phase was separated from the lower aqueous phase using a separatory funnel, and the aqueous phase was discarded. The solvent phase was evaporated, using a rotary evaporator (Buchi Rotovaper R-3, Flawil, Switzerland), resuspended in 1 mL ACN, and stored at −20 °C for later chemical analyses.

In brief: Grind leaves in water → Filter and collect leaf filtrate → Stir filtrate + solvents (50:50 EtOAc:hexane) → Separate aqueous and organic phases → Evaporate the organic phase containing OC and OA and resuspend in acetonitrile → Store for later analysis.

### 3.3. Privet Leaf-Extraction Protocol for 2 × 2 × 3 Factor Experimental Design

*L. vulgare* leaves were processed, either undried or fully dried at ambient conditions (apparent 0% water content [WC]). Leaves were ground in an aqueous solution buffered at either pH 8 or pH 6. The leaf slurry was then filtered and extracted with 50:50 EtOAc:hexane, either immediately (t = 0 min), after 15 min (t = 15 min), or after 1 h (t = 60 min), for a total of 36 extractions. The rest of the extraction followed the same steps as already described.

### 3.4. Privet Leaf Collections

*Ligustrum* leaves were collected in 2022 and 2023 from Bamboo Brook Outdoor Recreation Center and Willowwood Arboretum, situated 0.5 miles away from each other in Northern New Jersey (USA).

For the experiments presented in Table 1 and Figure 4, *L. vulgare* leaves were collected from two rows of 20-year-old hedges planted in parallel along a pedestrian path at Bamboo Brook. Branches were clipped and transported in a plastic bag to the lab, where, a few hours later, the top 10 pairs of leaves were pulled off by hand and stored at 5 °C for later extraction.

For the experiment presented in Table 2, leaves from *L. vulgare* (or common privet) and *L. ibolium* (or North privet) were collected on 27 September 2022, from six different 2-year-old plants of each cultivar, which were all planted in an isolated section of Willowwood arboretum. The same protocol, as described above, was applied for leaf collection.

For the experiment presented in Figure 3, leaves collected from eight *L. vulgare* plants in the same 2-year-old plant section of Willowwood arboretum were separated into two groups based on their emergence on the shoots: “younger leaves” corresponding to the first five to six emerging pairs of apical leaves, and “older leaves” corresponding to the pairs of leaves (up to six) just below on the same shoots. It is important to note that, at this latitude, the common privet is deciduous. Thus, all the leaves came from the new season. The leaf collections were performed on 18 July 2022, and again, from the same plants, on 27 September 2022.

For the experiment presented in Figure 5, leaves from *L. vulgare* and *L. obtusifolium* were collected at Bamboo Brook and those of *L. ibolium* at Willowwood. *L. ovalifolium* plants were obtained from Bay Gardens Nursery in East Moriches, New York, and *Olea europeae* Koroneiki (olive) plants came from Brighter Blooms Nursery in South Carolina. All leaves were collected and extracted in June 2023.

### 3.5. Liquid Chromatography Mass Spectrometry Analysis

#### 3.5.1. Stock Solutions and Calibration Standards

Stock solutions were prepared separately in ACN for OC (717 µg/mL) and OA (674 µg/mL), which were then combined and diluted in water to 100 µg/mL as an intermediate solution just prior to each calibration curve. From the intermediate solution, six calibration standard solutions were created by serial dilution in 90/10 water/ACN: 10, 5, 1, 0.5, 0.1, and 0.05 ng/mL. All calibration and sample solutions were prepared in polypropylene autosampler vials (ASVs) due to anecdotal data from our laboratory suggesting both OC and OA adhere to glass ASVs.

#### 3.5.2. Liquid Chromatography Mass Spectrometry

Ultra-high performance liquid chromatography (UHPLC) was performed on a ThermoScientific Vanquish Flex system, with a ThermoScientific Accucore Vanquish C18+ column (50 × 2.1 mm, 1.5 µm) (Waltham, MA, USA) that was maintained at 45 °C. A 5 µL injection volume was set with a 0.400 mL/min flow rate, using water with 0.1% FA for mobile phase A and ACN with 0.1% FA for mobile phase B, with the following gradient: initial %B was 10% and held for 0.5 min, linearly increased to 75% for 1.5 min, linearly increased to 95% for 3 min and held for 1.5 min, and linearly decreased to 10% for 1.5 min and held for 2 min.

Mass spectrometry was performed on a ThermoScientific Orbitrap Exploris 120 system (Waltham, MA, USA) operating at 60,000 resolving power in full-scan, negative-ion mode, with a scan range of *m*/*z* 50–700, and the RF lens at 70%. Ionization was achieved by heated electrospray at −2500 V and a vaporizer temperature of 350 °C. Quantitation was conducted on the [M−H]^−^ ion, for both OC and OA at *m*/*z* 303.1238 and *m*/*z* 319.1184, respectively.

### 3.6. Statistical Analyses

Arithmetic means were calculated across the replicates and were used for statistical analyses. Unpaired *t*-tests with Welch’s correction were performed in GraphPad Prism 6, and multifactorial ANOVA analyses were conducted in R version 4.4.0.

## 4. Conclusions

Our approach has been to investigate whether we could source OC and OA from plants belonging to the same family as the olive tree but found in abundance in many geographical regions and with less economic value. The results we obtained not only confirmed the previously reported presence of OC and OA in the leaves of the common privet (*L. vulgare*) but also demonstrated that, with an adapted treatment, the leaf extracts can serve as an excellent alternative source of both OA and OC, the latter being the more difficult of the two to obtain in large quantities. Specifically, in the presented method, the leaves are ground fresh (undried) in an aqueous solution and at room temperature. These conditions are favorable to enzymatic reactions. Most *Olea* leaf extracts in other studies are obtained using dried leaves, with methanol or other solvent solution maceration, or with hot aqueous solution maceration, particularly for the extraction of the glycosylated precursor oleuropein. Such conditions inhibit enzymatic reactions, which are required to produce OC and OA. In addition, we showed that leaf maceration at pH 6 and with longer time (after initial grinding, which decompartmentalizes enzymes) enrich further leaf extracts in OA and OC.

Our results also indicated that differences among privet species appear to have a stronger influence on OC and OA extract contents than the seasonal leaf-collection time or the leaf-developmental stage, although these findings will need to be confirmed with further investigations. The wide differences in OC and OA concentrations in leaf extracts of parent privet species, as shown in our work, are also noteworthy and may provide a useful point of departure in the study of their biosynthesis pathways.

## Figures and Tables

**Table 1:** Reproducibility of leaf-extraction method. Leaves were collected at the same time and area and were mixed in a plastic bag, from which 33 g of leaves were picked to produce one sample.

Extract	OC (mg/kg)	OA (mg/kg)
1	506.1	460.6
2	460.6	409.1
3	527.3	509.1
4	539.4	366.7
5	409.1	366.7
6	484.8	454.5
7	409.1	336.4
8	593.9	560.6
9	593.9	569.7
10	533.3	503.0
11	487.9	460.6
12	521.2	463.6
**Average**	**505.6**	**464.4**
**StdDev**	**60.0**	**69.5**
**RSD (%)**	**11.9**	**15.0**

**Table 2:** OC and OA concentrations (mg/kg) within and between leaf extracts of two *Ligustrum* species (*L. vulgare* and *L. ibolium*). Each extract came from one individual plant. Leaves were collected at the end of September 2022, on 2-year-old plants, all from the same location.

Extract	*Ligustrum vulgare*		*Ligustrum ibolium*	
	OC	OA	OC	OA
Plant 1	472.7	658.0	24.6	n.d.
Plant 2	445.5	469.7	108.8	11.8
Plant 3	490.9	630.3	81.8	8.6
Plant 4	302.1	521.2	90.3	5.9
Plant 5	345.5	503.0	45.8	4.2
Plant 6	312.1	238.2	48.5	3.1
**Average**	**394.8**	**503.4**	**66.6**	**5.6**
**StdDev**	**84.5**	**149.5**	**31.9**	**4.2**
**RSD (%)**	**21.4**	**29.7**	**47.9**	**74.7**

## Data Availability

Raw data are available per request from the authors.

## References

[1] Visioli F., Davalos A., López de las Hazas M., Crespo M.C., Tomé-Carneiro J. (2020). An Overview of the Pharmacology of Olive Oil and Its Active Ingredients. Br. J. Pharmacol..

[2] Ditano-Vázquez P., Torres-Peña J.D., Galeano-Valle F., Pérez-Caballero A.I., Demelo-Rodríguez P., Lopez-Miranda J., Katsiki N., Delgado-Lista J., Alvarez-Sala-Walther L.A. (2019). The Fluid Aspect of the Mediterranean Diet in the Prevention and Management of Cardiovascular Disease and Diabetes: The Role of Polyphenol Content in Moderate Consumption of Wine and Olive Oil. Nutrients.

[3] Soler-Rivas C., Espiń J.C., Wichers H.J. (2000). Oleuropein and Related Compounds. J. Sci. Food Agric..

[4] Michel T., Khlif I., Kanakis P., Termentzi A., Allouche N., Halabalaki M., Skaltsounis A.L. (2015). UHPLC-DAD-FLD and UHPLC-HRMS/MS Based Metabolic Profiling and Characterization of Different *Olea europaea* Organs of Koroneiki and Chetoui Varieties. Phytochem. Lett..

[5] Servili M., Sordini B., Esposto S., Urbani S., Veneziani G., Di Maio I., Selvaggini R., Taticchi A. (2014). Biological Activities of Phenolic Compounds of Extra Virgin Olive Oil. Antioxidants.

[6] Dinnella C., Masi C., Zoboli G., Monteleone E. (2012). Sensory Functionality of Extra-Virgin Olive Oil in Vegetable Foods Assessed by Temporal Dominance of Sensations and Descriptive Analysis. Food Qual. Prefer..

[7] Peyrot des Gachons C., O’Keefe A.J., Slade L., Beauchamp G.K. (2021). Protein Suppresses Both Bitterness and Oleocanthal-Elicited Pungency of Extra Virgin Olive Oil. Sci. Rep..

[8] Castejón M.L., Montoya T., Alarcón-de-la-lastra C., Sánchez-hidalgo M. (2020). Potential Protective Role Exerted by Secoiridoids from *Olea europaea* L. in Cancer, Cardiovascular, Neurodegenerative, Aging-Related, and Immunoinflammatory Diseases. Antioxidants.

[9] Karkovic Markovic A., Toric J., Barbari M., Brala C.J. (2019). Hydroxytyrosol, Tyrosol and Derivatives and their Potential Effects on Human Health. Molecules.

[10] Emma M.R., Augello G., Stefano V., Di Azzolina A., Giannitrapani L., Montalto G., Cervello M., Cusimano A. (2021). Potential Uses of Olive Oil Secoiridoids for the Prevention and Treatment of Cancer: A Narrative Review of Preclinical Studies. Int. J. Mol. Sci..

[11] Beauchamp G.K., Keast R.S.J., Morel D., Lin J., Pika J., Han Q., Lee C.H., Smith A.B., Breslin P.A.S. (2005). Ibuprofen-like Activity in Extra-Virgin Olive Oil. Nature.

[12] Costa V., Costa M., Videira R.A., Andrade P.B., Paiva-Martins F. (2022). Anti-Inflammatory Activity of Olive Oil Polyphenols—The Role of Oleacein and Its Metabolites. Biomedicines.

[13] Lozano-Castellón J., López-Yerena A., Rinaldi de Alvarenga J.F., Romero del Castillo-Alba J., Vallverdú-Queralt A., Escribano-Ferrer E., Lamuela-Raventós R.M. (2020). Health-Promoting Properties of Oleocanthal and Oleacein: Two Secoiridoids from Extra-Virgin Olive Oil. Crit. Rev. Food Sci. Nutr..

[14] Papakonstantinou A., Koumarianou P., Rigakou A., Diamantakos P., Frakolaki E., Vassilaki N., Chavdoula E., Melliou E., Magiatis P., Boleti H. (2023). New Affordable Methods for Large-Scale Isolation of Major Olive Secoiridoids and Systematic Comparative Study of Their Antiproliferative/Cytotoxic Effect on Multiple Cancer Cell Lines of Different Cancer Origins. Int. J. Mol. Sci..

[15] Nikou T., Sakavitsi M.E., Kalampokis E., Halabalaki M. (2022). Metabolism and Bioavailability of Olive Bioactive Constituents Based on In Vitro, In Vivo and Human Studies. Nutrients.

[16] Rivero-pino F. (2023). Oleocanthal—Characterization, production, safety, functionality and in vivo Evidences. Food Chem..

[17] Angelis A., Hamzaoui M., Aligiannis N., Nikou T., Michailidis D., Gerolimatos P., Termentzi A., Hubert J., Halabalaki M., Renault J.H. (2017). An Integrated Process for the Recovery of High Added-Value Compounds from Olive Oil Using Solid Support Free Liquid-Liquid Extraction and Chromatography Techniques. J. Chromatogr. A.

[18] Otero P., Garcia-Oliveira P., Carpena M., Barral-Martinez M., Chamorro F., Echave J., Garcia-Perez P., Cao H., Xiao J., Simal-Gandara J. (2021). Applications of By-Products from the Olive Oil Processing: Revalorization Strategies Based on Target Molecules and Green Extraction Technologies. Trends Food Sci. Technol..

[19] Jerman Klen T., Mozetič Vodopivec B. (2012). The Fate of Olive Fruit Phenols during Commercial Olive Oil Processing: Traditional Press versus Continuous Two- and Three-Phase Centrifuge. LWT.

[20] Martins B.T., Bronze M.R., Ventura M.R. (2022). Phenolic Compounds from Virgin Olive Oil: Approaches for Their Synthesis and Analogues. J. Agric. Food Chem..

[21] Oliverio M., Nardi M., Di Gioia M.L., Costanzo P., Bonacci S., Mancuso S., Procopio A. (2021). Semi-Synthesis as a Tool for Broadening the Health Applications of Bioactive Olive Secoiridoids: A Critical Review. Nat. Prod. Rep..

[22] Shimamoto Y., Fujitani T., Uchiage E., Isoda H., Tominaga K.I. (2023). Solid Acid-Catalyzed One-Step Synthesis of Oleacein from Oleuropein. Sci. Rep..

[23] Damtoft S., Franzyk H., Jensen R. (1993). Biosynthesis of Secoiridoid glucosides in Oleaceae. Phytochemistry.

[24] Gießel J.M., Zaar A., Schäfer R., Al-Harrasi A., Csuk R. (2018). Facile Access to Oleuropein and Hydroxytyrosol from *Ligustrum vulgare*—A Plant Material Growing All over Eurasia. Mediterr. J. Chem..

[25] Pieroni A., Pachaly P. (2000). An Ethnopharmacological Study on Common Privet (*Ligustrum vulgare*) and Phillyrea (*Phillyrea latifolia*). Fitoterapia.

[26] Czerwińska M.E., Gąsińska E., Leśniak A., Krawczyk P., Kiss A.K., Naruszewicz M., Bujalska-Zadrożny M. (2018). Inhibitory Effect of *Ligustrum vulgare* Leaf Extract on the Development of Neuropathic Pain in a Streptozotocin-Induced Rat Model of Diabetes. Phytomedicine.

[27] Nicolì F., Negro C., Vergine M., Aprile A., Nutricati E., Sabella E., Miceli A., Luvisi A., De Bellis L. (2019). Evaluation of Phytochemical and Antioxidant Properties of 15 Italian *Olea europaea* L. Cultivar Leaves. Molecules.

[28] Miho H., Díez C.M., Mena-Bravo A., Sánchez de Medina V., Moral J., Melliou E., Magiatis P., Rallo L., Barranco D., Priego-Capote F. (2018). Cultivar Influence on Variability in Olive Oil Phenolic Profiles Determined through an Extensive Germplasm Survey. Food Chem..

[29] Miho H., Moral J., Barranco D., Ledesma-Escobar C.A., Priego-Capote F., Díez C.M. (2021). Influence of Genetic and Interannual Factors on the Phenolic Profiles of Virgin Olive Oils. Food Chem..

[30] Ryan D., Prenzler P.D., Lavee S., Antolovich M., Robards K. (2003). Quantitative Changes in Phenolic Content during Physiological Development of the Olive (*Olea europaea*) Cultivar Hardy’s Mammoth. J. Agric. Food Chem..

[31] Koudounas K., Banilas G., Michaelidis C., Demoliou C., Rigas S., Hatzopoulos P. (2015). A Defence-Related Olea Europaea β-Glucosidase Hydrolyses and Activates Oleuropein into a Potent Protein Cross-Linking Agent. J. Exp. Bot..

[32] Obied H.K., Prenzler P.D., Ryan D., Servili M., Taticchi A., Esposto S., Robards K. (2008). Biosynthesis and Biotransformations of Phenol-Conjugated Oleosidic Secoiridoids from *Olea europaea* L.. Nat. Prod. Rep..

[33] Krakowska-Sieprawska A., Kiełbasa A., Rafińska K., Ligor M., Buszewski B. (2022). Modern Methods of Pre-Treatment of Plant Material for the Extraction of Bioactive Compounds. Molecules.

[34] Brogan A.P.S., Sharma K.P., Perriman A.W., Mann S. (2014). Enzyme Activity in Liquid Lipase Melts as a Step towards Solvent-Free Biology at 150 °C. Nature Com..

[35] Ghomari O., Sounni F., Massaoudi Y., Ghanam J., Drissi Kaitouni L.B., Merzouki M., Benlemlih M. (2019). Phenolic Profile (HPLC-UV) of Olive Leaves According to Extraction Procedure and Assessment of Antibacterial Activity. Biotechnol. Rep..

[36] Paiva-Martins F., Gordon M.H. (2001). Isolation and characterization of the antioxidant component 3,4-dihydroxyphenylethyl 4-formyl-3-formylmethyl-4-hexenoate from olive (*Olea europaea*) leaves. J. Agric. Food Chem..

[37] Onat S., Savaş E. (2019). Immobilization and Characterization of β-Glucosidase from Gemlik Olive (*Olea Europea* L.) Responsible for Hydrolization of Oleuropein. Ital. J. Food Sci..

[38] Ramírez E., Medina E., Brenes M., Romero C. (2014). Endogenous Enzymes Involved in the Transformation of Oleuropein in Spanish Table Olive Varieties. J. Agric. Food Chem..

[39] Takenaka Y., Tanahashi T., Shintaku M., Sakai T., Nagakura N., Parida (2000). Secoiridoid Glucosides from Fraxinus Americana. Phytochemistry.

[40] Czerwińska M.E., Ziarek M., Bazylko A., Osińska E., Kiss A.K. (2015). Quantitative Determination of Secoiridoids and Phenylpropanoids in Different Extracts of *Ligustrum vulgare* L. Leaves by a Validated HPTLC-Photodensitometry Method. Phytochem. Anal..

[41] Termentzi A., Halabalaki M., Skaltsounis A.L. (2015). From Drupes to Olive Oil: An Exploration of Olive Key Metabolites. Olive Oil Bioact. Const..

[42] Konno K., Hirayama C., Yasui H., Nakamura M. (1999). Enzymatic Activation of Oleuropein: A Protein Crosslinker Used as a Chemical Defense in the Privet Tree. Proc. Natl. Acad. Sci. USA.

[43] Breakspear I., Guillaume C. (2020). Molecules A Quantitative Phytochemical Comparison of Olive Leaf Extracts on the Australian Market. Molecules.

[44] Paiva-Martins F., Correia R., Félix S., Ferreira P., Gordon M.H. (2007). Effects of Enrichment of Refined Olive Oil with Phenolic Compounds from Olive Leaves. J. Agric. Food Chem..

